# Acute Pancreatitis With a Normal Serum Lipase in a Cystic Fibrosis Carrier

**DOI:** 10.7759/cureus.114558

**Published:** 2026-08-15

**Authors:** Rowena de Berker

**Affiliations:** 1 Internal Medicine, Wrightington, Wigan and Leigh NHS Foundation Trust, Manchester, GBR

**Keywords:** acute pancreatitis, cystic fibrosis (cf), cystic fibrosis transmembrane conductance regulator (cftr) protein, normal lipase pancreatitis, pancreatitis causes

## Abstract

We discuss a case of a young adult male presenting with radiologically confirmed acute pancreatitis, with a normal serum lipase throughout, confirmed to be a cystic fibrosis (CF) carrier. We discuss the sensitivity of lipase in acute pancreatitis and consider how acinar cell damage may lead to a normal lipase in individuals presenting with acute pancreatitis in the presence of an abnormal cystic fibrosis transmembrane conductance regulator (CFTR) gene. This case is unique in describing acute pancreatitis with a normal lipase in a patient with a single CF gene.

## Introduction

Acute pancreatitis is usually diagnosed with any two of the following three findings: (1) characteristic upper abdominal pain, (2) elevated pancreatic enzymes (typically >3 times the upper limit of normal), and (3) radiological evidence of acute pancreatitis [1]. The annual incidence of acute pancreatitis is estimated at 34 per 100,000 cases worldwide, and reported to be 29 per 100,000 cases in Europe [2,3]. The leading causes of acute pancreatitis are biliary obstruction due to gallstone disease and alcohol consumption [3]. Other causes, non-exhaustively, include hyperlipidemia, hypercalcemia, medication-induced pancreatitis, autoimmune pancreatitis, hereditary causes, and cases post-endoscopic retrograde cholangiopancreatography. Presentations can range from mild to a severe systemic inflammatory response with associated multi-organ dysfunction and increased mortality [4]. The World Society of Emergency Surgery states that 80-85% of people will have mild disease with a mortality rate of 1-3%; however, 20% will have moderately severe or severe disease with a mortality rate as high as 35% [5]. Local complications include pancreatic and peripancreatic necrosis, acute peripancreatic fluid collections, pseudocysts, and walled-off necrosis. Approximately 10% of acute pancreatitis cases will progress to become chronic pancreatitis [6].

Cystic fibrosis (CF) is a genetically recessive condition. Individuals with carrier status have a single mutation of the cystic fibrosis transmembrane conductance regulator (CFTR) gene. This gene encodes an ion channel responsible for transport of chloride and bicarbonate across epithelial membranes, including in the lungs and pancreas [7]. Multiple studies suggest that CF carriers, who have a single recessive gene variant and, by definition, do not have the disease, are at increased risk of a range of conditions associated with CF, including pancreatitis [8]. The prevalence of acute pancreatitis in cystic fibrosis is dependent on the CFTR variant; milder variants are more likely to develop acute pancreatitis on the basis of retaining sufficient pancreatic tissue and function; one cohort study described a prevalence of 17.3% for acute pancreatitis in a subgroup with maintained pancreatic sufficiency [9].

## Case presentation

Patient information

A male in his late teens presented with a four-day history of acute-onset left upper quadrant pain. The pain was described as constant, maximal at onset, "gripey" in nature, and unaffected by eating. There was associated vomiting, with no fever or diarrhea. The patient disclosed an alcohol binge of approximately 30 units of alcohol the evening prior to the onset of symptoms. He reported on average one binge of alcohol per week, outside of which alcohol consumption was reported as minimal, and he did not smoke tobacco. Notable medical history included congenital right renal agenesis and Nissen fundoplication for gastroesophageal reflux disease (GERD) (with two subsequent revisions for persistent reflux associated with vomiting, most recently four years prior to presentation). He took no regular medications and had no significant family history.

Clinical findings

On examination, the abdomen was soft with mild left upper abdominal discomfort, without peritonism or guarding, and bowel sounds were normal. Baseline observations were normal.

Diagnostic assessment

**Figure 1:**
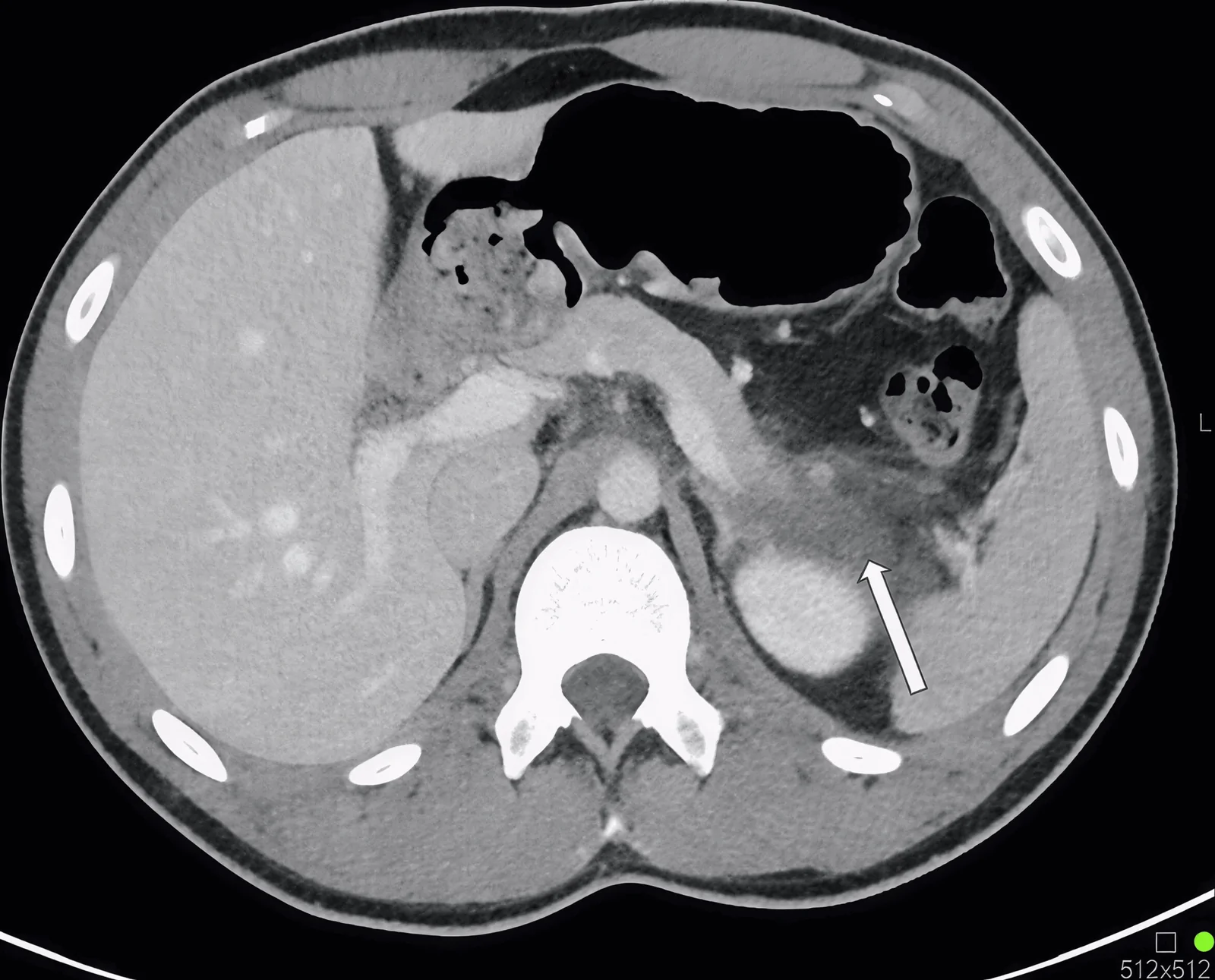
CT scan demonstrating localized inflammatory stranding and trace fluid surrounding the pancreatic tail. The arrow points to the area of inflammatory stranding and trace fluid around the pancreatic tail.

Bloods were grossly normal, other than a mildly elevated C-reactive protein (CRP). The serum lipase was 12 U/L, which is notably at the very lower limit of the normal reference range of 10-60 U/L (Table 1). The case was discussed with the general surgical team, who recommended a computed tomography (CT) scan. The CT demonstrated localized inflammatory stranding and trace fluid surrounding the pancreatic tail, in keeping with acute pancreatitis (Figure 1).

**Table 1:** Laboratory blood test results. ALT: alanine transaminase; AST: aspartate transaminase; ALP: alkaline phosphatase; eGFR: estimated glomerular filtration rate

Test	Result	Normal range
ALT	17 U/L	0-40 U/L
AST	20 U/L	10-50 U/L
ALP	76 U/L	30-150 U/L
Bilirubin	7 µmol/L	2-20 µmol/L
Albumin	37 g/L	32-48 g/L
Lipase	12 U/L	10-60 U/L
Hemoglobin	148 g/L	130-175 g/L
WBC	9.2x10^9^/L	4-11x10^9^/L
Sodium	138 mmol/L	135-145 mmol/L
Potassium	4.0 mmol/L	3.5-5.2 mmol/L
Creatinine	95 µmol/L	50-110 µmol/L
eGFR	99 mL/min/1.73 m^2^	80-120 mL/min/1.73 m^2^
CRP	23 mg/L	<5 mg/L
Total cholesterol	4.2 mmol/L	<5 mmol/L
Triglycerides	0.5 mmol/L	<2.3 mmol/L
Corrected calcium	2.4 mmol/L	2.2-2.6 mmol/L

Therapeutic intervention

The patient was admitted under general surgery, and a repeat serum lipase remained normal (18 U/L) at approximately 48 h after the first result. Abdominal ultrasonography showed no gallstones and a clear common bile duct, with a normal diameter (3 mm), which strongly excluded gallstone etiology. The Glasgow-Imrie score for severity of acute pancreatitis was 0, indicating a low risk of severe pancreatitis [10]. The patient improved with intravenous fluids and simple analgesia; he was discharged after 24 h of admission under the general surgical team.

Follow-up and outcomes

Subsequent outpatient genetic testing was performed as part of the workup for acute pancreatitis. This confirmed that the patient was a carrier of a cystic fibrosis gene variant. The patient has had no further episodes of abdominal pain and remained well at the time of follow-up.

## Discussion

The presumed trigger for the development of acute pancreatitis in this case was alcohol. Case reports exist describing normal lipase in acute pancreatitis, and indeed an elevated lipase is not required to fulfill the diagnostic criteria. The novel features of this case are how the patient being a carrier for CF may have contributed to his development of acute pancreatitis and resulted in a normal lipase. Lipase has been cited to have a higher sensitivity than amylase, with a longer half-life and, therefore, an extended diagnostic window [11]. Lipase levels rise within 2 h of insult and remain elevated for up to two weeks [12]. This patient presented within the established diagnostic window for lipase elevation in acute pancreatitis.

There is increasing awareness that carriers of CF are at an increased risk of various CF-associated conditions, such as pancreatitis [8]. The mechanism for increased risk of pancreatitis in the presence of a defective CFTR gene is complex. It is understood that in cystic fibrosis there is impaired hydration of luminal contents, impaired pancreatic ductal secretion of bicarbonate, altered pH, and the potential sequelae of ductal inflammation, obstruction, and fibrosis [7,13]. Structural changes such as fibrosis are also a feature of chronic pancreatitis, in which it is an established norm that pancreatic enzyme levels are often normal [14]. However, in this case, CT did not show evidence of chronic pancreatitis, and the patient's clinical history was not suggestive of chronic pancreatitis. Patients with two copies of an abnormal CFTR gene can have low serum lipase levels due to exocrine insufficiency [15]. The author suggests that a possible hypothesis for the normal serum lipase in this case is that being a carrier of a single CFTR gene may have led to a degree of exocrine insufficiency or acinar cell damage. It is hoped that this study can contribute to emerging literature and research in this area. In summary, the cause of acute pancreatitis in this case was likely multifactorial, with the environmental trigger of an alcohol binge and a background of genetic predisposition contributing to the unique presentation. The author undertook a scoping review to contextualize this case report within the existing literature and could not identify any case reports describing acute pancreatitis with a normal serum lipase level in a patient with cystic fibrosis carrier status.

## Conclusions

We found this to be a thought-provoking case that considers the spectrum of disease that can present in a CFTR carrier, and we believe it contributes to the growing body of knowledge in this area. This case provides a compelling reminder that serum lipase cannot be relied upon exclusively to exclude acute pancreatitis and raises the possibility of why lipase may be more likely to remain normal in a patient with an abnormal CFTR gene. Clinicians should maintain a high level of clinical suspicion and consider the overall clinical picture rather than relying heavily on a single test to exclude a diagnosis.
